# Eye Movement Training and Suggested Gaze Strategies in Tunnel Vision - A Randomized and Controlled Pilot Study

**DOI:** 10.1371/journal.pone.0157825

**Published:** 2016-06-28

**Authors:** Iliya V. Ivanov, Manfred Mackeben, Annika Vollmer, Peter Martus, Nhung X. Nguyen, Susanne Trauzettel-Klosinski

**Affiliations:** 1 Vision Rehabilitation Research Unit, Centre for Ophthalmology, University Eye Hospital, Eberhard-Karls University of Tuebingen, Tuebingen, Germany; 2 ZEISS Vision Science Lab, Institute for Ophthalmic Research, Centre for Ophthalmology, Eberhard-Karls University of Tuebingen, Tuebingen, Germany; 3 The Smith-Kettlewell Eye Research Institute, San Francisco, United States of America; 4 Low Vision Clinic, Centre for Ophthalmology, University Eye-Hospital, Eberhard-Karls University of Tuebingen, Tuebingen, Germany; 5 Institute for Clinical Epidemiology and Applied Biometry, Eberhard-Karls University of Tuebingen, Tuebingen, Germany; University of Lincoln, UNITED KINGDOM

## Abstract

**Purpose:**

Degenerative retinal diseases, especially retinitis pigmentosa (RP), lead to severe peripheral visual field loss (tunnel vision), which impairs mobility. The lack of peripheral information leads to fewer horizontal eye movements and, thus, diminished scanning in RP patients in a natural environment walking task. This randomized controlled study aimed to improve mobility and the dynamic visual field by applying a compensatory Exploratory Saccadic Training (EST).

**Methods:**

Oculomotor responses during walking and avoiding obstacles in a controlled environment were studied before and after saccade or reading training in 25 RP patients. Eye movements were recorded using a mobile infrared eye tracker (Tobii glasses) that measured a range of spatial and temporal variables. Patients were randomly assigned to two training conditions: Saccade (experimental) and reading (control) training. All subjects who first performed reading training underwent experimental training later (waiting list control group). To assess the effect of training on subjects, we measured performance in the training task and the following outcome variables related to daily life: Response Time (RT) during exploratory saccade training, Percent Preferred Walking Speed (PPWS), the number of collisions with obstacles, eye position variability, fixation duration, and the total number of fixations including the ones in the subjects' blind area of the visual field.

**Results:**

In the saccade training group, RTs on average decreased, while the PPWS significantly increased. The improvement persisted, as tested 6 weeks after the end of the training. On average, the eye movement range of RP patients before and after training was similar to that of healthy observers. In both, the experimental and reading training groups, we found many fixations outside the subjects' seeing visual field before and after training. The average fixation duration was significantly shorter after the training, but only in the experimental training condition.

**Conclusions:**

We conclude that the exploratory saccade training was beneficial for RP patients and resulted in shorter fixation durations after the training. We also found a significant improvement in relative walking speed during navigation in a real-world like controlled environment.

## Introduction

“Tunnel vision” is a severe concentric vision loss in the periphery of the visual field (VF), a condition often caused by retinitis pigmentosa (RP). Peripheral vision, despite its low resolution, is important for creating and updating an accurate representation of surrounding space for navigation [1]. Therefore, RP is a condition that impairs mobility, as demonstrated by Turano and colleagues [1] in a memory-guided walking task. This functional impairment has been further confirmed by complementary findings of differences in eye movement scanning strategies in patients with tunnel vision and healthy observers [2].

The rationale for this study is based on two established facts: It has been shown in a variety of paradigms that scanning eye movements are led by attention [3,4]. Secondly, there are two mechanisms of attention that may be relevant here: A “sustained” component that is slow, voluntarily controlled and does not depend on visual input, and a “transient” component that is fast, reflex-like, and depends on visual input [5,6]. This is why transient attention is based on, “bottom-up” processing of (exogenous) information, while sustained attention is a mechanism that uses goal-oriented “top-down” (endogenous) commands [5–12]. For example, salient stimuli can attract transient attention, even though the subject had no intention to attend to these stimuli [7,8]. On the other hand, if visual input is missing or compromised, as e.g. by a retinal disease like RP, the transient and stimulus-driven mechanism does not work any more. In this case, goal-directed “sustained” attention [5–12] can be voluntarily directed to objects and features in the seeing visual field, and even to locations in unseen regions in space. Thus, we can ask subjects to make eye movements using sustained (i.e. “top-down”) attention, which is slow and consciously controlled, independent of visual input. The question ensuing from this is whether both these mechanisms can play a role for the objectives of the current study.

The question of whether top-down or bottom-up processing is deployed for eye movement control depends on the spatial distribution of seeing and non-seeing portions of the visual field. This was demonstrated by Vargas-Martin and Peli, who instructed subjects with RP to walk among and avoid obstacles, while their eye movements were recorded [2]. They reported that scanning eye movements in RP patients are reduced compared to healthy controls and suggested that this could be accounted for by the lack of peripheral information. This finding can be interpreted as evidence for control of eye movements by “bottom-up” processing. However, this does not rule out the possibility that top-down processing may be involved if RP patients are subjected to different experiments, using visual search in a laboratory and real-world walking environments [13]. The results from this study [13] suggested that the role of attention is task-specific and that attention can be voluntarily deployed outside the seeing visual field. In the search task, subjects looked for “pop-out” targets presented on a projection screen at different eccentricities outside their VF, while in the real-world experiment they walked freely through an unfamiliar indoor environment and along city streets. In these conditions, patients with tunnel vision made a surprisingly high number of saccades beyond their restricted visual field, with a frequency similar to that observed in healthy observers. About two thirds of the 107 saccades/min in the visual search and one third of the 56 saccades/min in the walking experiment landed beyond the VF [13]. In another study [14], participants with glaucoma were asked to locate a 4 deg target, pseudo-randomly selected within a 26 × 11 deg natural image. Interestingly, some participants made a few eye movements into the blind visual field, while others did not move their eyes outside their seeing VF at all [14].

Several studies have investigated the effectiveness of training to make exploratory saccades into the blind field in patients with homonymous hemianopia (HH) [15–18]. In contrast to tunnel vision in RP, HH is a condition characterized by vision loss in half of the VF, which disrupts the normal scanning eye movement strategies. Also, HH differs from RP in that vision loss is caused by damage to higher visual regions of the brain, rather than due to a condition in the retina. It has been shown that patients with HH can be trained to make systematic scanning saccades into their blind hemi-field. This training consequently led to an enlargement of their dynamic visual field, i.e. the region, in which subjects can successfully locate a target by eye movements [15, 16, 18]. More importantly, there was a transfer of functional benefit to daily life activities, such as natural search and exploration on the blind side. This demonstrates that top-down information processing is involved in successful vision rehabilitation. Similar results were obtained by Kuyk and colleagues who studied the effect of exploratory saccade training (EST) in a large cohort of patients with vision loss [19]. They found that patients' performance in exploratory tasks improved with training and led to fewer collisions with obstacles under dim conditions. However, only two out of their 78 patients had been diagnosed with RP while the majority had age-related macular degeneration, which mainly affects central vision and causes fewer mobility problems than RP.

The aim of the present study was to find out whether the benefits of exploratory saccade training found for other visual field deficits extend to RP. To assess the benefits, we measured oculomotor responses during walking and when avoiding obstacles in a controlled environment. Additionally, this study could provide further insight into the mechanism of eye movement control in situations requiring proactive eye movement control. We tentatively hypothesize that top-down spatial and bottom-up object-oriented attention can play an important role in directing eye movements while trying to avoid obstacles that fall inside and outside the seeing VF.

We subjected RP patients to EST in a randomized controlled trial. Patients had a large range of ages and disease durations. To assess the effectiveness of training, we used outcome variables that are relevant for everyday life. Our expectation was that eye movement training in RP patients would result in a reduction of response times in the assigned training task. We also looked for a transfer in functional benefits to daily life, by measuring the percent preferred walking speed and number of collisions with obstacles and patients' eye movement scanning strategies before and after training, in a controlled real-world environment. In addition, effects on the patients' quality of life were assessed by questionnaire before and after training. To control for any placebo effects of having training per se, we also had a control group, who received reading training that used rapid serial visual presentation (RSVP): The patients had to fixate the center of a computer screen, where a series of words that had to be recognized appeared one at a time. Due to the lack of eye movements, this text presentation technique is unlikely to evoke visual search behavior and can be considered as placebo training.

## Materials and Methods

### Ethics

The study was approved by the ethics committee of the University of Tübingen Medical Faculty, and informed written consent was obtained from all participants. The research adhered to the tenets of the Declaration of Helsinki.

### Subjects

In a waiting list control group design, subjects were randomly assigned to experimental training (saccade group, n = 14) or control training (reading group, n = 11). For ethical reasons and to ensure that all patients had a chance to benefit from the study, the patients, who were first assigned to the reading group, afterwards also underwent experimental training (waiting list group). Additionally, data from 10 healthy, normally-sighted observers were included as a non-training control group. Below, these will be referred to as “healthy control group”. This group did not receive training but underwent the same tests as the patient groups over the same period of time. Thus, the healthy control group reveals the degree of performance change that would occur without training. The disclaimer here is that it would only reveal this for healthy controls. All healthy controls had normal or corrected to normal vision. The RP groups did not differ regarding age, diagnosis, or duration of disease (for details see Demographics and S1 Table). The inclusion criterion for the study was RP with visual acuity higher than 0.1 dec (1.0 logMAR) and maximum visual field diameter of less than 30 deg. Patients with coexisting eye movement pathologies or cognitive impairments were excluded. In the 25 participating RP patients, who complied with the task to train for the prescribed period, we recorded reaction time (RT) and walking speed (WS). Eye movements were recorded in only 14 RP patients (7 in the saccade training group, 5 in the reading training and 2 from the waiting list). Eye tracking failed in another 11 patients, because of issues with calibration. Because of the advanced stage of their disease, prior to taking part in the experiments, all RP patients, at some point in their life, had experienced orientation and mobility (OM) training to learn how to maximize their useful vision. None of them used a guide dog in daily life, only five patients occasionally used long canes (see S1 Table, patient numbers 7, 10, 11, 20, 24).

### Demographics

The saccade (mean age of 53,5 +/- 12.5 SE years) and reading (mean age of 51,2 +/- 11 SE years) training groups did not differ regarding age. For the saccade group, mean duration of disease (Dur), visual acuity (VA), visual field (VF) and magnification (Mag) were 31.4, 0.3, 19.6, 2.1, respectively. Mag is the magnification factor required by the patients for reading normal print size. VF is the horizontal diameter [deg] of the binocular visual field (measured monocularly and then superimposed). VA is the binocular visual acuity in logMAR. Duration (Dur) of the disease and age of the patients are given in years. For the reading group Dur = 26.2, VA = 0.3, VF = 19.6, Mag = 2.0, while for the waiting list Dur = 27.8, VA = 0.3, VF = 18.5, Mag = 2.0.

### Eye Tracking

The Mobile Tobii Glasses Eye Tracker (version 1) was used to continuously record the subjects' eye position while walking in our mobility course. The glasses perform video-based eye tracking using the dark pupil and corneal reflections, as well as a scene camera operating at 30 Hz with 56 x 40 degrees scene coverage. Prior to each trial, a system-guided 9-point calibration was performed until the highest possible tracking quality was achieved. The Tobii glasses are a light and unobtrusive IR tracking and scene recording system that provides monocular recordings (right eye) with no distracting cameras or mirrors. The recording unit is small (like a mobile phone) and allows the subject to move freely without having to carry bulky or heavy equipment. The field of view allowed by the glasses is enough for navigation and slightly larger than the field of view used in the experimental saccade training condition. It is also significantly larger than the patients' largest intact visual field (30 degrees diameter). However, the field of view allowed by the Tobii glasses is significantly smaller than the normal field of view, almost 180-degree forward-facing horizontal diameter. Eye tracking quality was assured by accepting only trials with less than 30 percent loss of eye position coordinates.

### Exploratory Saccade training

**Fig 1:**
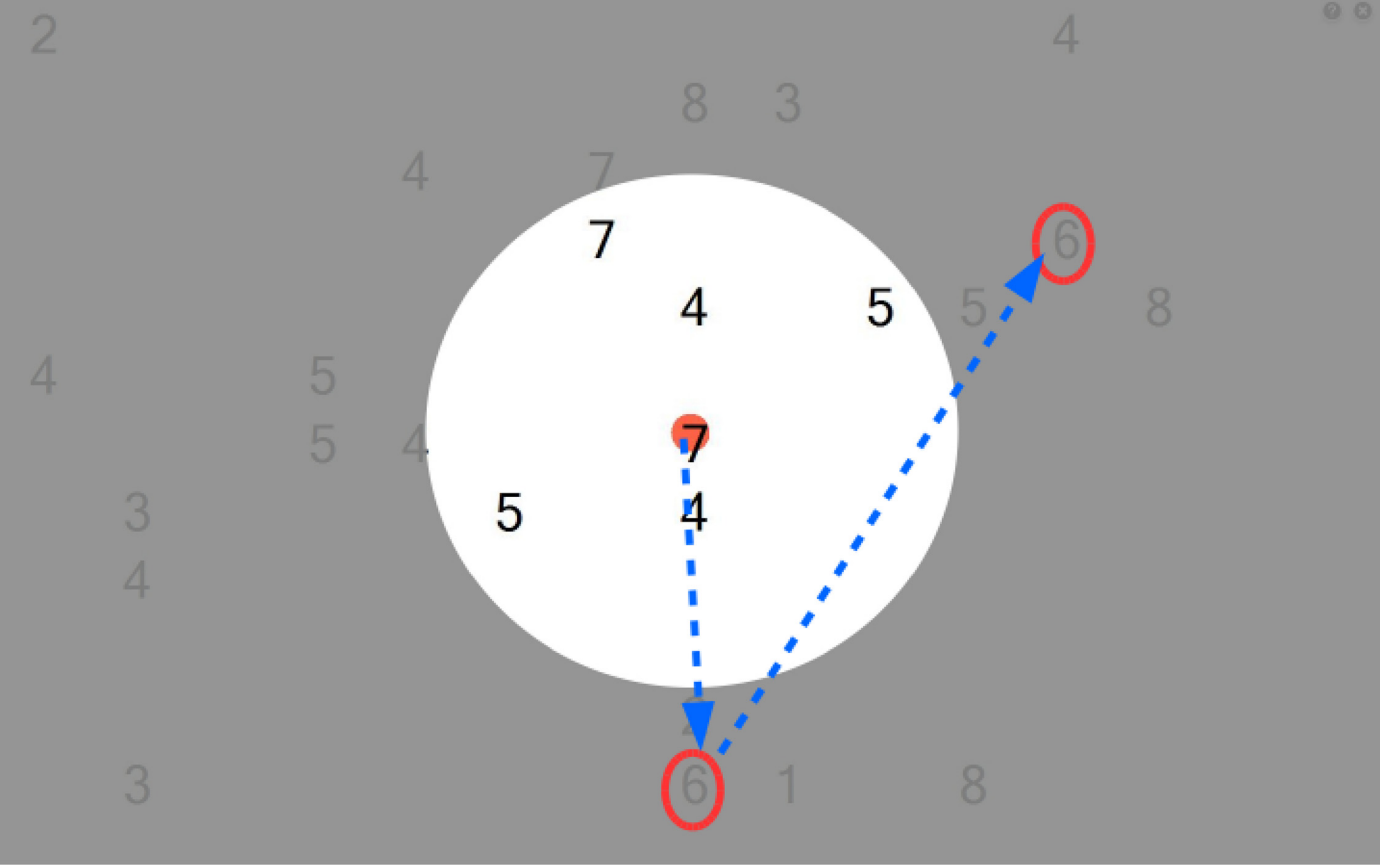
A schematic illustrating the visual search training task. In this screen shot, the targets (number 6) are embedded among a random array of digits as distracters. It was possible that multiple targets simultaneously appeared on the screen. After initial fixation of the central red dot, subjects were required to perform saccades (indicated by the blue arrows) outside their seeing visual field (gray area) to find all the targets (in the schematic surrounded by the red ovals). Each screen was presented until all targets were detected. The time-out period between the different screens was determined by the subjects.

EST was implemented as a saccadic search task that aimed to improve visual search outside the seeing VF and the use of the total field of gaze. A schematic illustrating the visual search training task is given in Fig 1. The task was practiced on a laptop computer placed 30 cm from the patients’ eyes (total visual field 35 deg × 47.7 deg). Custom software was used to generate a random array of digits or letters (0–9; A-Z; size adjusted to the individual patient’s need) distributed with equal probabilities on the blind and seeing parts of the VF. RP subjects had to find and move the mouse pointer over the predefined digit or letter (for example, digit 6). Upon passing over the digit, the program generated a beep, recorded the time it took the subject to find the single target (RT per target) and turned it into a red symbol, which provided positive feedback and prevented double search for the digit. After finding all digits, the time between the initial screen onset and last target found was recorded (RT per screen). The screen was then automatically cleared, and the patient started the next trial by clicking a button centered on the screen, which ensured initial central fixation for the next trial. Position and RTs for all digits found per screen were stored in a database for each daily session. Initially, patients came to our laboratory and were instructed how to perform the training. The RP subjects then practiced at home. Detailed description of this method is given in Roth et al. 2009 [18].

### Reading Training

In the (control) reading training task, we used rapid serial visual presentation (RSVP). This technique presents words sequentially, one single word at a time, at the same fixated location on the screen instead of presenting a full page of text. For a detailed description of the method see [20–22]. In contrast to the saccade training, this task can here be considered as placebo training since fixation of a single word in the center of the screen eliminates eye movements towards the periphery. For both training methods, the size of the digit to be found (saccade training) as well as of the text (reading training) was adjusted according to the individually required magnification for each subject.

#### Data collection protocol

In both training conditions (saccadic and reading) the subjects trained at home, using our laboratory’s laptop computers to ensure standard training conditions (screens, fixed viewing distance, and visual field area trained), twice per day for 30 minutes, 5 days a week, for 6 weeks. Patients were instructed to avoid head movements during training. Each computer was equipped with a distance indicator that assisted subjects in maintaining the correct viewing distance. In the saccade training, RT data were collected twice (before and after the training), while no RTs were recorded during reading training. The analysis of the automatically saved log-in files indicated whether the patients of both groups complied with the training protocol. After completing the reading training, the group also underwent saccade training (waiting group). Three patients who took part at T1, T2 and T3 in the reading group did not wish to continue participating in the experiments. Data from these patients were included in the reading group for the analysis but not in the waiting group, which included 8 patients only.

### Mobility course

The mobility course was a 68 m long and 1.3 m wide hallway containing 30 obstacles that were arranged in pseudo-random positions to ensure an approximately equal amount of obstacles on the left and right side. After the initial randomization, the configuration was kept throughout the course of the study. To assess any effect of training that transfers to functional benefits in real life, we measured percent preferred walking speed (PPWS) and eye position variability before and after training. Walking speed was measured in meters/second, while the mobile eye tracker (Tobii glasses) was used to record eye positions while walking in the standard mobility course, closely following the design of the one by Black and colleagues [23]. While subjects walked the course, we also counted the number of errors that were defined as the number of contacts with obstacles. The obstacles were single horizontal (40 x 40 x 60 cm) or vertical (60 x 40 x 40 cm) and double (120 x 40 x 40 cm) cardboard boxes. They were empty and not fixed to the floor, which minimized the danger for the patients to fall and hurt themselves. Subjects had to walk the whole course under two illumination conditions: photopic (in the following called “light”) and mesopic (in the following called “dim”). The average illumination in the *light* condition was 65 cd/m^2^, while in the *dim* condition was 5 cd/m^2^. Subjects were instructed to walk through the course as fast and accurately as possible, while maintaining their safety and avoiding to run. Walking speed data were collected at three times: pre- (T1) and post- (T2) training, as well as at 6 weeks after the training (follow-up at T3). Subjects walked in the hallway without obstacles, in both *light* and *dim* conditions, prior to the obstacle courses. All 4 conditions (light and dark, with and without obstacles) were performed on a single day. The healthy control group conducted the same walks on two separate days: runs with obstacles in the *light* and *dim* conditions, as well as obstacle-free walks in the *light* and *dim* conditions. We compared performance of the healthy control group in the two runs to ensure that just performing the mobility task of walking along the course itself did not improve performance. Our obstacle and obstacle-free conditions were designed to simulate different walking conditions that might occur in a natural environment: bright light (photopic), or in low light (mesopic), cluttered or free of obstacles. In contrast to freely walking outdoors, where all these conditions can be intermingled, we could clearly separate the different conditions in our controlled environment. Hence, we could differentiate between typical eye movement strategies and potential training effects in the different conditions. No canes were used during these experiments.

### Outcome variables

#### Response times in the visual search task

We collected all RTs per screen for each subject and each daily session, and averaged them to give us the RTs per session. Furthermore, we calculated the mean response times of the first and last three RTs per session to compare the subject’s mean RTs before and after saccade training.

#### Mobility

Percent preferred walking speed. We measured the preferred walking speed (*PWS*) obtained during walking the obstacle-*free* path and the walking speed (*WS*) during walking the course *with* obstacles. We then calculated the percent preferred walking speed (*PPWS*), a measure of relative slowing [24], according to the following formula: *PPWS = WS / PWS x 100*. The *PPWS* is used here, because it is an objective measure of mobility performance that allows valid inter-subject comparisons by accounting for individual characteristics such as age and physical condition of each subject. Moreover, the use of the PPWS is useful for our study because Clark-Carter and colleagues [25] have shown that it allows the use of smaller samples in experiments by reducing inter-subject variations.

Eye-movement analysis. We calculated the total number of fixations and average fixation durations for each subject and each intervention (saccade or reading training). We categorized the fixations made by the patients outside their intact visual field (saccades into blind areas). The detection was performed using a velocity-based algorithm for saccade detection proposed by Engbert and Kliegl [26]. The algorithm labels those eye movement episodes as saccades that show a velocity exceeding a certain threshold. Anything between two saccades is considered a fixation. A similar algorithm, based on a velocity threshold detecting method, was shown to adequately detect saccades in data collected by a mobile eye tracking device [27]. We also compared horizontal and vertical eye position variabilities of the RP patients in the different experimental groups with the healthy control group. Variability was calculated separately for the horizontal and vertical eye position components. We consider the variabilities (standard deviations of the horizontal and vertical eye positions) a useful outcome variable, because they provide a measure of the spatial range of eye movements while walking [2].

Quality of life. Quality of life was assessed by the German version of NEI-VFQ 25 questionnaire at the pre-, post- and follow-up training. The questionnaire included the following categories with one to six items each: general health; general eyesight; eye pain; near vision; distance vision; vision-specific impact on social functioning; vision-specific impact on mental condition; vision-specific effects on social role behavior; vision-specific effects on dependence on others; driving; color vision; peripheral vision.

#### Statistical analysis

We compared the response times between the first and last three days of training on the EST task by a 2 sample t-test. The outliers in the RT distribution were removed before computing the means by the interquartile range (IQR) rule. The IQR is the length of the box in a box-and-whisker plot. Briefly, according to the IQR rule, an outlier is any value that lies more than one and a half times the length of the box from either of its ends. Statistical differences between groups were assessed using pairwise t-tests, provided that normality assumptions were met. PPWS in *light* and *dim* conditions was analyzed by a mixed model repeated measures (MMRM) ANOVA with time before (T1) and after (T2) training as within subject factor and group (saccade and reading training) as between subjects factor. This analysis was applied only to the saccade and reading training groups, since not all patients in the waiting list control group were able to participate in the subsequent saccade training. Non-training controls were also excluded from the primary MMRM ANOVA analysis, since they differ by diagnosis (no disease) and treatment (no treatment). However, controls were used to show baseline differences from subjects with disease, which justifies the chosen paradigm, and to show stability of measurements over time. Furthermore, we excluded the measurement after six weeks (T3) from this analysis, since not all subjects who took part in the study participated (4 subjects did not participated in T3) in the follow-up phase. Thus, including the T3 would lead to unbalanced layout of our analysis. Therefore, *light* and *dim* conditions were analyzed with two separate 2-way ANOVAs, since the main research question addressed by this analysis is whether performance would change with training. Performance differences under different lighting conditions are to be expected and not of primary interest here. However, since a combined model should also be considered relevant to our design, we performed a 3-way ANOVA with combined light and dim conditions as additional within-subject factor, which reached the same conclusions as with the separate ANOVAs. One-way ANOVAs were used to compare means from total number of fixations and average fixation duration for the three groups, saccade training, control reading training and non-training healthy controls separately in the *light* and *dim* conditions. Only significant interactions are discussed here.

To determine whether normal distributions could be assumed for the eye position variability data, Q-plots and Lilliefors two-sided normality tests were performed separately for each group: healthy controls and RP-reading or RP-saccade training patients. For all obstacle and obstacle-free training conditions, in the pre- and post- phases, we analyzed the horizontal and vertical components of the eye position variability separately. The hypothesis of normality was rejected for all eye position variability distributions. Hence, we used the Kruskal-Wallis test and the Wilcoxon signed rank test for all comparisons between training groups and conditions. Unless otherwise stated, data from the waiting group were not combined with the saccade training group for the statistical analysis. We used R (version 2.15.1), a language and programming environment for statistical computing, for the data analysis [28]. Descriptive statistics and analysis of covariance were used to analyze the NEI-VFQ 25 questionnaire. However, p-values and significances are not confirmatory as no correction for multiple testing was applied due to low power.

## Results

### Response times in the visual search task

All subjects in the saccade and waiting training groups were trained to perform a visual search task outside their seeing VF. Statistical analysis was performed only on data from patients (n = 25) who complied with the task to be trained for the prescribed period. We found for all analyzed subjects that the RTs significantly decreased (p<0.05) between the start and the end of the training task. The mean RT for the first 3 days of training was 7.5 s +/- 1 SEM while it was 4.7sec +/- 2 SEM for the last 3 days (six weeks in between).

### Percentage of preferred walking speed

**Fig 2:**
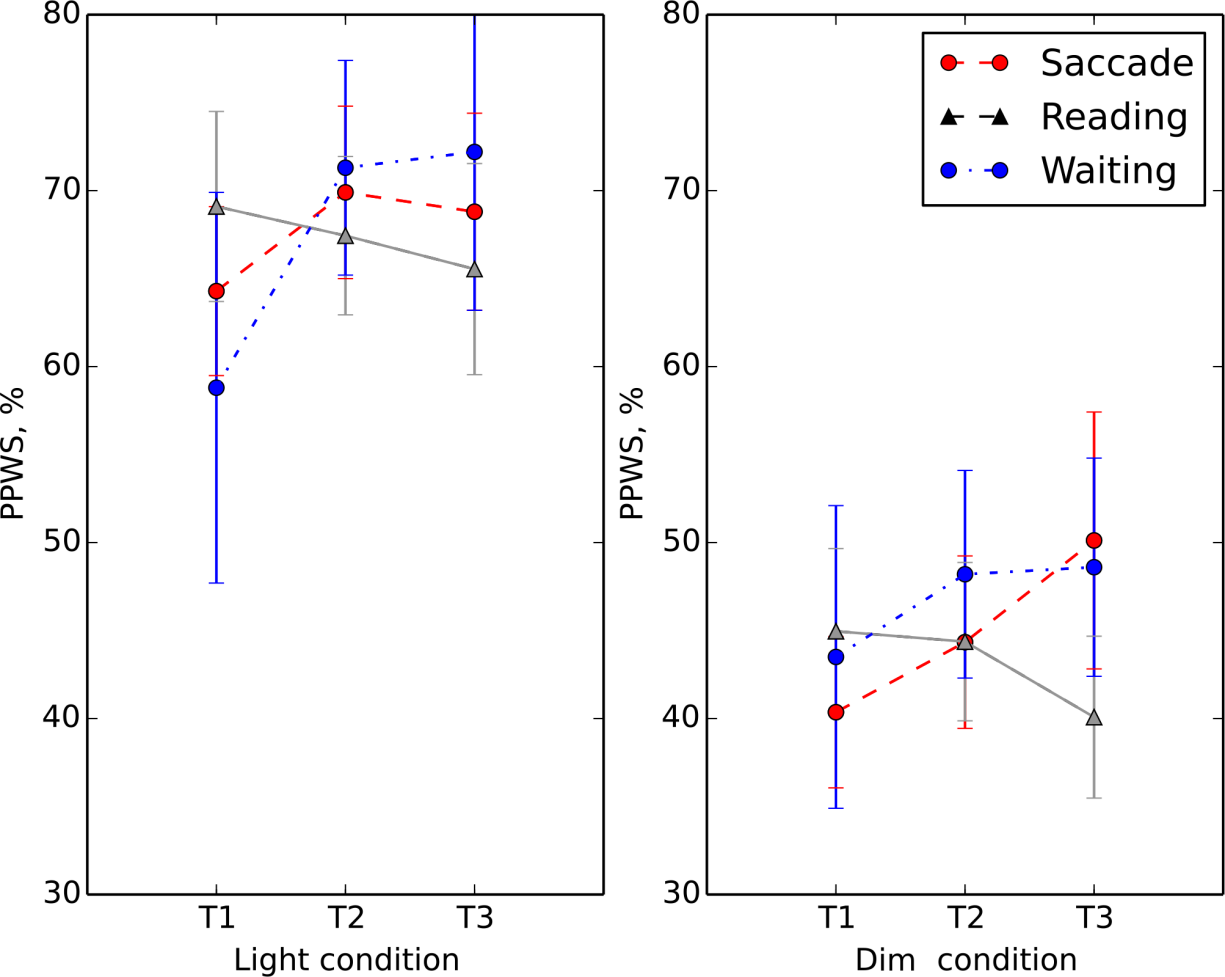
Separate plots present the light (left plot) and dim (right plot) conditions for the saccade (red circles), reading (gray triangles) and waiting list (blue circles) training groups. The average percentage of preferred walking speed (PPWS) is plotted on the ordinate as function of the different times relative to the training–pre (T1)-, post (T2)- and follow-up (T3). Error bars represent +/-1 SEM.

Average PPWS for the healthy control subjects was 92%, which was higher than for any of the RP subjects groups: saccade training (59%), and reading training (55%). Individual PPWS for the patients in the different training groups are given in S1 Table. Pairwise t-tests showed that PPWS at T1 and T2 did not change in either the healthy (non-training) or reading control groups (p>0.05 and p>0.05, respectively). Fig 2 shows the PPWS for the different training groups (saccade, reading and waiting list) before and after training (T1 and T2) in the different lighting conditions. In the *light* condition we did not find time to be a significant factor (F = 3.12, p = 0.09), while the interaction “time” x “group” was significant (F = 6.99, p = 0.015). A simple contrast for the within subjects factor group revealed that time (T1 and T2) was a significant factor only in the saccade training group (F = 9.89, p = 0.008). This interaction indicates that walking speed in the saccade training group improved more after training than in the reading group. In the *dim* condition, time was not a significant factor (F = 0.810, p = 0.4), nor was the interaction “time” x “group” (F = 1.066, p = 0.3).

We analyzed the average number of errors pre- and post-training made by the saccade and reading training patients and found that it was significantly higher (p < .001.) in the *dim* (number of errors n = 12) than in the *light* condition (number of errors n = 3). The number of errors in the pre- and post- training phase for the different groups did not change significantly: The saccade training patients made on average 7 errors before and 6 errors after training (p = 0.1), while the reading training group made on average 8 errors before and after training.

We were interested whether the improvement in walking speed after training in the *light* condition for the saccade training group could be predicted by the age, visual acuity or the size of residual visual field of the subjects. Therefore, we fitted a multiple linear regression model with PPWS as response variable and age, visual acuity and the size of residual visual field of the subjects as explanatory variables. By eliminating the non-significant terms, we found a significant regression equation (F (1, 30) = 14.8, p < .001.), with an adjusted r^2^ of 0.31. The patients’ predicted PPWS was 34.6 + 3.2 (VF) + 0.0 (VA) + 0.0 (A), where VA is visual acuity, VF is the size of the intact visual field and A is the age of subjects. The significant coefficient in the equation above (p < .001.) represents the mean change in the response PPWS for one unit (deg) of change in the predictor VF.

### Number of fixations and fixation duration

**Fig 3:**
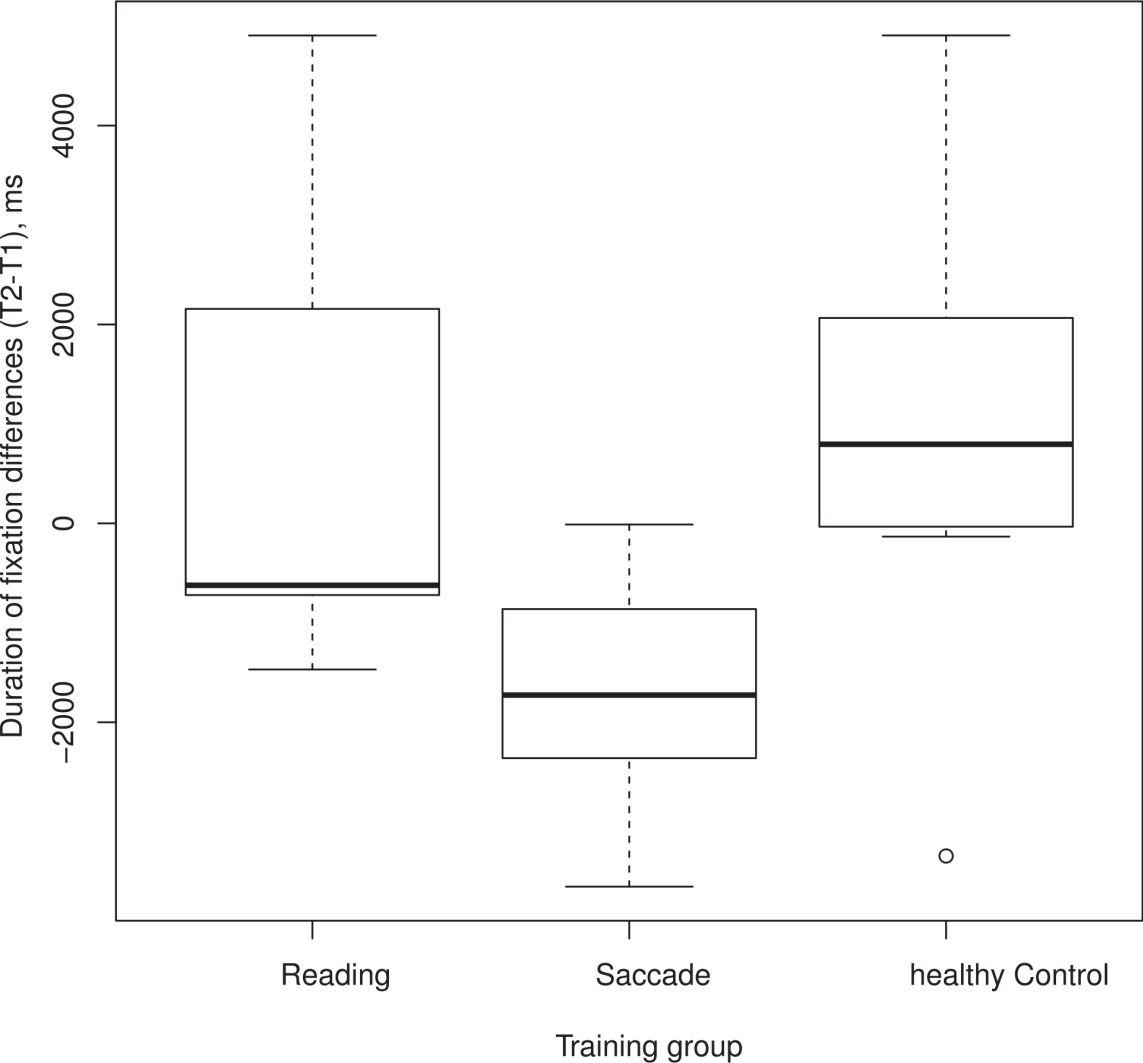
Box plot comparison of patients’ eye movements with healthy control observers before (T1) and after (T2) training. The abscissa represents the three training groups (reading, saccade or healthy control). The ordinate shows the duration of fixations for each training group, calculated as differences between the durations of fixations performed after and before training (T2-T1). Negative numbers indicate that fixation durations were shorter after training.

Table 1 lists the frequencies of the fixations per minute, the average fixation duration, and the number of fixations beyond the VF in RP patients and the healthy control group. In both *light* and *dim* conditions, a one-way ANOVA did not reach significance when the differences (T2-T1) for the number of fixations done in the saccade training group was compared with that of the reading training and healthy control groups. This finding shows that the number of fixations that subjects in either of the training groups performed was not influenced by the training. For the fixation duration parameter in the *light* condition (see Fig 3), the differences (T2-T1) for fixation durations performed by the saccade training group are compared with both the reading training and healthy control groups. The plot demonstrates that saccade training is effective and that only the subjects who were in the saccade training group made shorter fixations after training. A one-way ANOVA showed that the difference was significant (F = 4.119, p = 0.03), but not for the *dim* condition (F = 2.19, p = 0.14). Table 1 also shows that before and after training, the RP subjects in the reading and saccade training groups, on average, directed more than 30 percent of their saccades to a region outside of the intact VF.

**Table 1:** Fixation durations (FD, in s), frequencies of fixations (FF, in fixations per minute) and number of fixations outside the seeing VFs (N_out_, in fixations per minute) of patients with RP in the reading and saccade training groups. Performance in the light and dim experimental conditions is shown before (T1) and after (T2) training.

Light condition				Dim condition			
Group	FF (T1/T2)	FD (T1/T2)	Nout (T1/T2)	Group	FF (T1/T2)	FD (T1/T2)	Nout (T1/T2)
saccade	39.8/47.9	2.8/1.1	16.3/19.7	saccade	31/32.9	3.5/ 1.77	8.6/9.2
reading	47.01/60	1.14/1.95	20.0/21.4	reading	55.8/28.2	1.9/2.8	17.5/11.4
healthy control	31.9/20.1	2.1/ 3.05		healthy control	37.3/17.2	3.12/ 3.7	

### Eye position variability

**Fig 4:**
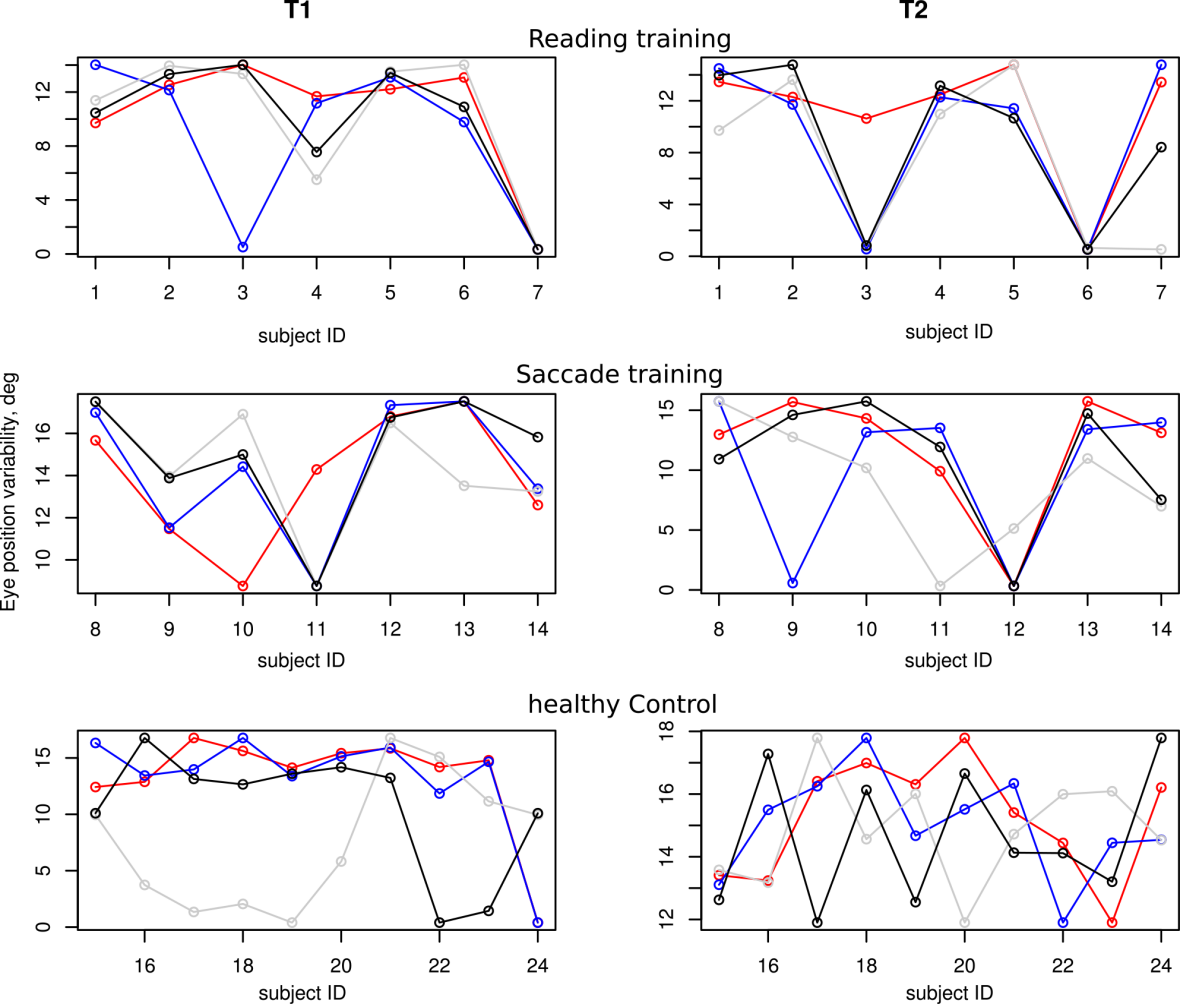
Horizontal eye position variability [degrees] pre- (T1) and post-training (T2) for the different training conditions: reading, saccade and healthy control groups on the vertical axes, while the horizontal axes represent different observers. Blue circles represent the light condition, red the dim condition, and black and gray the values for the two walks without obstacles(S2 Table provides a table with all values along the horizontal and vertical axes). In most patients (saccadic and reading group) the eye position variability is in a similar range as in the healthy observers.

We calculated the eye position variability (standard deviations of horizontal and vertical eye positions) separately for each of the different training conditions at T1 and T2 (shown in Fig 4).

**Fig 5:**
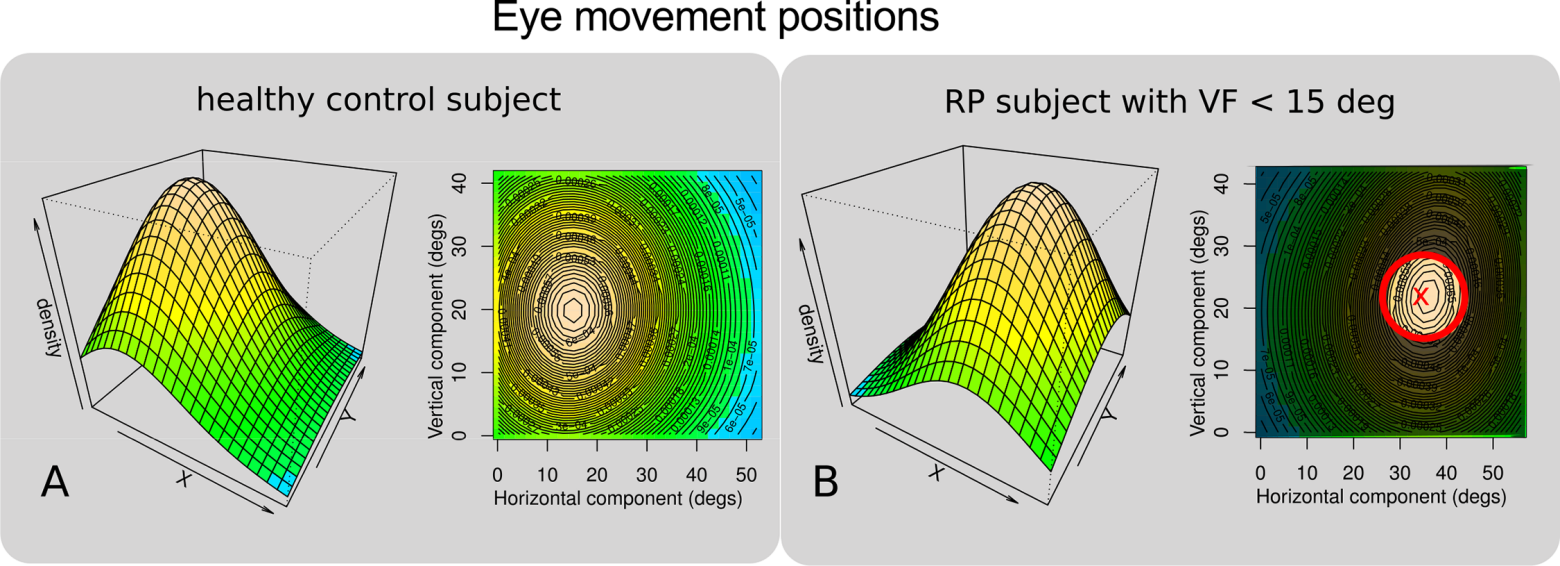
Perspective and contour plots of two-dimensional kernel density estimations of horizontal and vertical eye movement positions. Eye positions (horizontal and vertical on the abscissa and ordinate, respectively) and visual field horizontal diameters are given in degrees. Density represents the time the eye spent at any particular position (x,y) during the whole walk and are given in percentages. In 5A typical eye movement positions of a normal observer in the light condition are shown. In 5B are shown the typical eye movement positions of an RP patient (visual field depicted by the red circle surrounded by the shaded gray area) in the light condition.

Fig 5 shows the perspective and contour plots of two-dimensional kernel density estimations of the raw horizontal and vertical eye movement positions for a typical normally sighted subject (5A) and a representative RP patient (5B), in the *light* condition. This figure gives intuition about the subjects' range of eye movements (x and y coordinates) and time spent (density estimation) at a particular position during the whole walk. Note that the tracker coordinate system is used as a reference frame in the graph.

**Fig 6:**
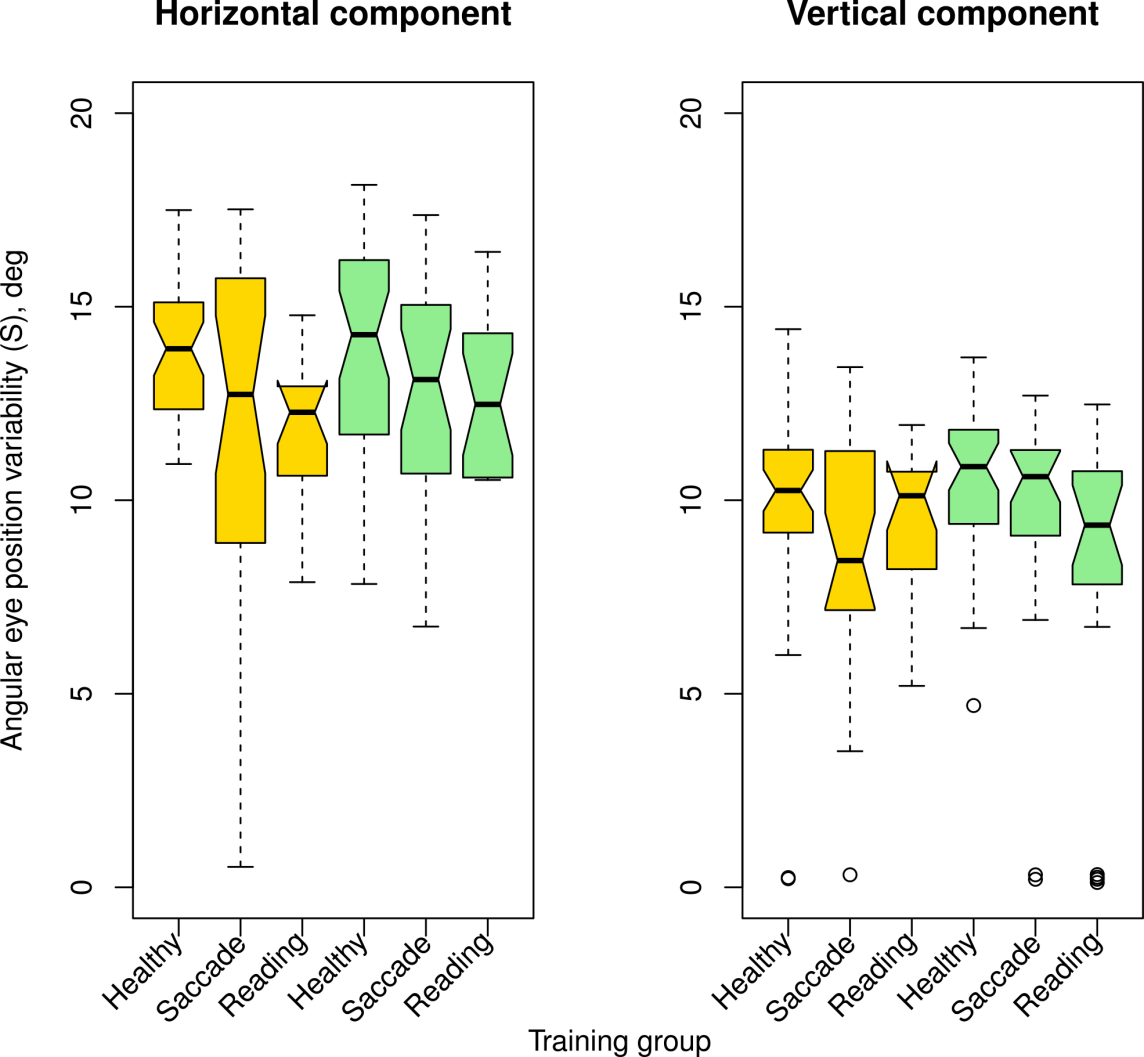
Box plots of horizontal eye position variability in degrees. The horizontal component is plotted on the left, the vertical component is shown on the right. Different training groups are plotted on the abscissa for the pre-training (gold symbols) and post-training (green symbols) conditions. Boxes represent the median and the 25th to 75th percentile of the distributions; whiskers represent 1.5* IQR excluding outliers. This shows that the range of horizontal eye movements amplitudes was significantly larger than that of the vertical eye movements.

The left plot in Fig 6 displays the horizontal component of eye position variability for pre- (gold symbols) and post- (green symbols) training in the different training groups. Similarly, the vertical component of pre- and post-training variability are shown on the right. Note that horizontal variability (left plot) is larger than the vertical one (right plot) for all three groups, healthy controls and patients with saccade and reading training.

For all healthy control observers, the range of horizontal eye movements (Fig 6, left plot) was significantly larger than that of the vertical eye movements (Fig 6, right plot) in the different conditions, (p < 0.001.). Interestingly, we found that the same eye movement range (horizontal variability > vertical variability) was predominant in most of the RP subjects, both at T1 and T2. For all training groups (saccade and reading), the range of horizontal eye movements was significantly larger than that of the vertical eye movements (horizontal vs. vertical eye position variability compared, p < .001.). Paired T-tests on the horizontal vs. vertical variability showed that horizontal and vertical components were not significantly different in the condition without obstacles (p > 0.05 at both, T1 and T2 for the saccade and reading training groups), while this was not true for the condition with obstacles (p < 0.05 at both, T1 and T2 for the saccade and reading training groups).

### Quality of life

On average, the quantitative analysis by the German version of NEI-VFQ 25 questionnaire at pre-, post- and follow-up training did not show any differences. However, 5 RP patients reported after completing the saccade training that they felt better, that they benefited from the training, and that they had improved in regard to some of the questionnaire questions.

## Discussion

This study investigated whether we can demonstrate that RP patients can benefit from exploratory saccade training (EST) by studying eye movements and by using outcome variables that relate to everyday life. Additionally, we tested whether EST that encourages RP subjects to make systematic scanning saccades into the blind visual field would result in enlargement of their dynamic VF. We used a reading training task as a control condition, which used rapid serial visual presentation, which is unlikely to elicit visual search behavior. We also compared eye movement strategies by comparing eye position variability in RP patients with that of healthy control subjects.

We found a significant reduction in response times (37%) for the saccade training group, which corresponds well with previous findings in subjects with hemianopia [16–18]. Roth and colleagues demonstrated that exploratory eye movement training led to improved performance of their patients in a natural search task in the affected hemifield. In the natural search task, RT was measured using common objects presented in irregular but fixed positions on a table, where patients had to find a particular object. The reduced RTs in the present study further demonstrate that EST can be used as a general technique to improve eye movement behavior. We also found that walking speed increased significantly only for the saccade training group. The increase demonstrated in our study (6%), is small compared to the benefit of 23% demonstrated in the natural search task by Roth and colleagues [18]. However, the improvement of PPWS was in the range of that found in the study by Soong and colleagues [29], where only short term improvement of 6% in PPWS was demonstrated by mobility training. Taking these studies together, the results demonstrate that improvements of eye movement behavior observed after EST training transfer to functional benefits in real life and may be specific to the task and the amount of visual field loss.

We found that the RP subjects in the reading and saccade training groups, in both *light* and *dim* conditions at T2, directed more than 30 percent of their saccades, on average, to a region outside of the intact VF (see Table 1). This also holds for the average number of saccades made already before training (T1) in both *light and dim* conditions. This high number of saccades outside the seeing visual field is not surprising and agrees with the findings of Luo et al. [13], whose RP subjects made a similar percentage (33%) of saccades outside of the intact VF in a visual search task. Together, these results suggest that, if a saccade into a non-seeing part of the visual field is necessary, top down processing can govern eye movement control. The number of fixations, for both *light* and *dim* conditions, did not differ significantly before and after training, neither in the saccade nor in the reading training group. This finding shows that the number of fixations that subjects in either of the training groups performed, was not influenced by the training. However, in the *light* condition and only in the saccade training group, the fixation duration was shorter after training. This demonstrates that exploratory saccade training is effective in patients with RP, so that their eye movements become more effective while walking and avoiding obstacles.

### Walking performance

It has been shown that people who become familiar with the walking route by practice require less mental effort to navigate in this environment, so that improved efficiency (higher PPWS) and a higher degree of safety (reduced number of errors) can be observed [30]. We found that, on average, RP patients performed worse than healthy control subjects, which suggests that our walking task demanded a high degree of mental effort from them and that the chosen paradigm is sensitive to the disease. A comparison between 2^nd^ and 1^st^ examination in healthy control subjects shows that the results do not change considerably (+2.4%) by just practicing the task. Together with the fact that patients improve with saccade training (+5.6%) but not with reading training (-2.6%), this supports the hypothesis that the improvement effect is specific to the training. The finding that the PPWS in the RP patients improved only for the saccade training group immediately after training (T2) and after six weeks (T3), but not in the reading training group, also suggests that improvement through mere repetition is unlikely (Fig 2). Fig 2 also shows that the PPWS for the saccade training group remains elevated even six weeks after training as measured at follow-up, while in the reading training group, PPWS did not improve after the training or at follow-up and remained at the initial level. Moreover, there was also no improvement in regard to safety in either of the training groups, which indicates that the subjects did not become familiar with the course by repetition, as we would then expect fewer collisions. This finding is in contrast with the findings by Kuyk et al. [19], where walking speed did not improve after EST training in AMD patients, who were assumed to have normal peripheral vision. However, the subjects in the current study significantly reduced the number of collisions with obstacles only in the *dim* walking conditions. Altogether, these results demonstrate empirically that the benefits are specific to the kind of intervention and the disease, since only the RP group assigned to the exploratory eye movement training improved in the walking course task.

The most frequent mobility problems in people with RP include walking more slowly than normally sighted people, fear of falling, collisions with objects, difficulty with changing lighting levels, and bumping into people. Hence, these patients feel less comfortable traveling alone [31,32]. The small increase in PPWS observed here can only be a partial functional benefit for walking in the real world, as a significant reduction in the number of collisions due to better scanning was not observed. Furthermore, some RP patients reported that they feel better and benefited from the training, although our results by the descriptive statistics used in the questionnaire regarding the quality of life on average did not show a significant improvement.

### Top-down visual processing determines eye movement strategies in our context

We expected a change of eye movement scanning range due to exploratory saccade training—a functional benefit for the patients that would demonstrate an increased use of consciously controlled “sustained” attention to control eye movements through top-down information processing. Interestingly, we found that the eye movement strategies of all RP patients in most training conditions were similar to those of the healthy control observers already before training, which is in contrast to previous findings [2]. Therefore, we could not observe any effect of the saccade training on the range of amplitudes of eye movements.

Our finding that the RP patients made a high number of fixations outside their intact VF is consistent with the finding of comparable horizontal and vertical eye position variability in healthy control subjects. It is also consistent with the findings of Luo et al. [13], where RP patients also performed many saccades (33%) outside of the intact VF in a visual search task. Taken these findings together, our results point towards top-down control of exploratory eye movements into blind parts of the VF, where consciously directed attention is an important prerequisite in eye movement planning and execution. Further, these findings suggest that the conscious planning of eye movements are task- and context-specific. Our analysis of the variability of the RP saccade and reading training groups under different task conditions further confirms the conclusion about different eye movement strategies under different task conditions: We found that horizontal and vertical eye position variability is not significantly different in the condition without obstacles, while this was not true for the condition with obstacles.

### Importance of eye movements for navigation

Previous research investigated eye movement control by either analyzing saccades (amplitude, number) or eye position variability and led to two contradicting conclusions regarding the question whether eye movement control is driven by bottom-up or top-down information processing. A possible explanation emerges if the characteristics of different types of attention are considered. If only saccades within the seeing VF are investigated, then the reflex-like “transient” attention based on bottom-up processing is sufficient. The resulting saccades rapidly shift the image of a peripheral target to the fovea. On the other hand, saccades to a point in space that is not in the seeing part of the VF need to be initiated by a conscious effort, which can be achieved by using “sustained” attention [5]. Consequently, the resulting saccades show a longer latency due to the temporal characteristics of this attentional mechanism.

### Limitations of this study

It should not be forgotten that people suffering from RP will solve some of their problems regarding mobility in the real world by a combination of eye and head movements. In such cases, the attentional mechanisms described here will be invoked as well as the vestibulo-ocular reflex that may partially counteract an eye movement to maintain fixation. On the other hand, the possibility of goal-directed head movement training should also be considered. Hence, the current investigation can only be viewed as a first step in an attempt to fully understand the interplay of eye and head movements in these patients while navigating in the real world. Hence, the main findings of this study should be interpreted with caution. Although our experimental environment resembled the real world, it was still limited compared with the full range of challenges that RP patients face in everyday life. For instance, our mobility course did not include changes in elevation or instantaneous changes in illumination (light adaptation), which is a severe challenge for RP patients: RP imposes night blindness and problems with glare. This can cause great difficulty with light adaptation when moving from the shadow of a building to bright sunlight, or walking into or out of a building on a sunny day. Furthermore, patients with advanced RP have problems with changes in elevation and reduce their scanning of the environment, as they tend to look down a lot because they are afraid of falling. Therefore, future studies on patients' performance in an environment with mixed illumination and elevation changes will be necessary to show the extent to which exploratory saccade and head movements training can be beneficial for the daily life of RP patients.

## Supporting Information

S1 Table Overview of subject data: Patient demographics and percent preferred walking speed (PPWS) calculated at PRE-, POST- and FOLLOW-UP in light and dim conditions for the different training groups: saccade (S), reading (R), waiting list (W) and healthy controls (H). Mag is the magnification factor required by the patients for reading normal print size; VF is the horizontal diameter [deg] of the binocular visual field (measured monocularly and then superimposed); VA is the binocular visual acuity in logMAR. Duration (Dur) of the disease and age of the patients are given in years. Underlined numbers denote the number of errors (collisions with obstacles) for each condition. Means for each group and subjects whose eye movements were recorded are printed bold.
(CSV)

S2 Table Eye position variability pre- and post-training for the light, dim and free walk conditions. Eye position variability is given for the horizontal/vertical component separately (X/Y) in degrees of visual angle.
(CSV)

S3 Table Eye movement analyses on number of fixations and fixation duration. TD—total duration (ms); NF—number of fixations; DF—duration of fixation; NF._out_- number of fixations outside the seeing visual field; VF.d—diameter of the seeing visual field (deg); Lcond -light condition: l = light, d = dim.
(CSV)
